# Improving Accuracy and Temporal Resolution of Learning Curve Estimation for within- and across-Session Analysis

**DOI:** 10.1371/journal.pone.0157355

**Published:** 2016-06-15

**Authors:** Matthias Deliano, Karsten Tabelow, Reinhard König, Jörg Polzehl

**Affiliations:** 1 Department Systems Physiology of Learning/AG Brain-Machine-Interfaces, Leibniz Institute for Neurobiology, Magdeburg, Germany; 2 Research Group Stochastic Algorithms and Nonparametric Statistics, Weierstrass Institute for Applied Analysis and Stochastics, Berlin, Germany; 3 Special Lab Non-Invasive Brain Imaging, Leibniz Institute for Neurobiology, Magdeburg, Germany; University of Zurich, SWITZERLAND

## Abstract

Estimation of learning curves is ubiquitously based on proportions of correct responses within moving trial windows. Thereby, it is tacitly assumed that learning performance is constant within the moving windows, which, however, is often not the case. In the present study we demonstrate that violations of this assumption lead to systematic errors in the analysis of learning curves, and we explored the dependency of these errors on window size, different statistical models, and learning phase. To reduce these errors in the analysis of single-subject data as well as on the population level, we propose adequate statistical methods for the estimation of learning curves and the construction of confidence intervals, trial by trial. Applied to data from an avoidance learning experiment with rodents, these methods revealed performance changes occurring at multiple time scales within and across training sessions which were otherwise obscured in the conventional analysis. Our work shows that the proper assessment of the behavioral dynamics of learning at high temporal resolution can shed new light on specific learning processes, and, thus, allows to refine existing learning concepts. It further disambiguates the interpretation of neurophysiological signal changes recorded during training in relation to learning.

## Introduction

Learning, the acquisition of knowledge through experience, manifests as behavioral changes in the course of training. Learning behavior relies on a multitude of neural and cognitive processes which act on different spatial and temporal scales [1–3]; however, many of these processes are not accessible experimentally. Therefore, any particular learning experiment is influenced by numerous uncontrolled variables. This entails a certain degree of unaccountable variability of behavior across time, within a subject as well as between subjects [4]. As a consequence, single behavioral responses of individual subjects are difficult to interpret with respect to learning.

In order to relate changes in behavioral performance to learning, statistical analyses across trials and subjects are indispensable. In conventional learning curve analyses behavioral performance is analyzed in moving trial windows. At any instant in time performance is quantified by the success probability, i.e. the probability of a correct response, estimated as the proportion of correct trials among all *n* trials of a window. The window size is adjusted to the particular interest of the experimenter and may range from a few trials to entire sessions. Learning is then identified from the time course of the resulting learning curve as an increase of the estimated success probability [5].

This ubiquitous approach to the analysis of learning entails some serious statistical problems. First, it relies on the assumption that the success probability is constant within the moving window. Deviations from this assumption cause estimated success probabilities to be biased. The severity of the effect depends on the chosen window size that also determines the precision and accuracy of the estimates. Depending on the individual learning speed, changes of success probabilities will be faster or slower, and therefore require shorter or longer analysis windows to preserve stationarity. This makes it difficult to choose an adequate window size before the analysis.

Furthermore, as many learning tasks extend over long periods of time, training is often carried out in several consecutive sessions with sometimes prolonged breaks between them. This introduces discontinuities in the learning process, e.g. by memory consolidation during inter-session sleep [6, 7]. To avoid analysis problems, learning is often considered only on the session level assuming constant performance within a session. Performance changes within the session are thereby not considered relevant for learning, but regarded as a disturbing factor in performance estimation that has to be controlled experimentally [8]. However, Gallistel and colleagues [9], for example, have demonstrated that performance changes within a session are not just a confounding factor, but are systematically related to learning.

Second, even with a proper choice of window size, learning curves display a high inter-subject variability [10]. Statistical analysis of learning curves is therefore predominantly carried out on the population level by means of artihmetic averaging of learning curves across subjects [11]. However, it is commonly neglected that the grand-mean learning curve is the mean of Bernoulli probabilities bound between 0 and 1 estimated across discrete trials, and that standard errors of the mean are not well suited to display the variability of such a quantity.

In this work, we frame the analysis of learning curves as the statistical problem of estimating the Bernoulli probability of a correct response in a trial based on the observed responses in a small (n < 200) trial window centered over that trial. We propose different statistical methods designed for estimating these success probabilities from small samples, which also provide more appropriate confidence intervals than the conventional analysis. These methods are evaluated in comparison with the conventional analysis using simulated and experimental data derived from an exemplary learning experiment in which rodents were trained in a two-way active avoidance paradigm in a shuttle-box [4, 12]. Evaluation showed that moving window estimation of learning curves is prone to various random and systematic errors depending on learning phase, window size, and the statistical model employed.

To better account for *systematic* errors (bias) arising from a non-stationary success probability in the analysis window, we applied a generalized linear model (GLM) [13], which allows the success probability to vary systematically across trials in the analysis window. Furthermore, we used a Firth’s penalized likelihood approach [14] to estimate the parameters of the GLM. This adjusts for biases in case of small or large probabilities and guarantees that estimates will be finite, opposite to the conventional likelihood estimate. Optimal window sizes balancing variability and systematic errors were derived by minimizing cross-validated estimates of the mean squared error of prediction. To retain discontinuities in the learning process introduced by session breaks, learning curves were estimated separately for each training sessions. Finally, population learning curves and their confidence intervals were estimated with a fixed effects model, which is statistically more adequate than conventional population averaging. Taken together, these methods significantly improved the accuracy and temporal resolution of learning curve analysis both for single-subject and population analysis.

## Materials and Methods

### Learning curve analysis

#### Estimation of individual learning curves

For each trial *i* = 1, …, *n* of a training session, the response of a subject in the learning process can be regarded as a Bernoulli variable *Y*_*i*_ ∼ *B*(1, *p*_*i*_) describing a correct (*Y*_*i*_ = 1) or an incorrect (*Y*_*i*_ = 0) response. The probability *p*_*i*_ of correct responses is a measure of learning performance for each trial. The time course of this success probability *p*_*i*_ over trials is called a learning curve.

To estimate individual learning curves, we employed two different models, a *constant model* and a *generalized linear model.* In the constant model, the success probability was estimated by the binomial proportion of correct responses within a window *W*_*i*_ of bandwidth *h* centered at trial *i* including the *h* preceding and the *h* succeeding trials. Success probability was assumed to be constant within *W*_*i*_. The constant model underlies the conventional moving window analysis (from now on referred to as constant model (conv)), in which the window is moved across trials irrespective of session breaks; hence, potential discontinuities at the session boundaries are ignored. However, abrupt, step-like performance changes across session breaks cannot be modelled by a conventional windowing approach. To account for such discontinuities of the learning process between sessions, we performed the moving window analysis separately for each training session (termed constant model (sep) in this work), i.e. the window was *not* moved across session breaks. For equidistant trial times *t*_1_, … *t*_*n*_ within a session, this window encompasses the subset of trials given by

$$W_{i}=\left\{j|\max\left(t_{1},t_{i}-h\right)\leq t_{j}\leq \min\left(t_{i}+h,t_{n}\right)\right\}.$$ (1)

The size of the window, i.e. the number of trials #*W*_*i*_ within the window, is given by #*W*_*i*_ = min(*t*_*i*_ + *h*, *t*_*n*_) − max(*t*_1_, *t*_*i*_ − *h*) ≤ 2 * *h* + 1 depending on the bandwidth *h*. In case the assumption of a constant model is met, the maximum likelihood success probability for trial *i* can be estimated by means of the binomial proportion of correct responses in *W*_*i*_:

$${\hat{p}}_{i}=\sum_{j\in W_{i}}Y_{j}/\#W_{i}.$$ (2)

Many of the commonly applied statistical inference methods approximate the binomial distribution of this estimated success probability by a Gaussian distribution. Thus, standard statistics textbooks (see, e.g., [15]) recommend the use of confidence intervals with nominal coverage probability (1 − *α*) (i.e. the probability that the confidence interval contains the true value) for the evaluation of the success probability, based on asymptotic normal approximations given as

$$CI\left(p_{i}\right)=\left({\hat{p}}_{i}-\kappa \sqrt{\frac{\hat{p_{i}}\left(1-\hat{p_{i}}\right)}{\#W_{i}}},{\hat{p}}_{i}+\kappa \sqrt{\frac{\hat{p_{i}}\left(1-\hat{p_{i}}\right)}{\#W_{i}}}\right),$$ (3)

where *κ* is the (1 − *α*/2)-quantile of the standard Gaussian distribution, and the term $\sqrt{\hat{p_{i}}\left(1-\hat{p_{i}}\right)/\#W_{i}}$ is an estimate of the standard deviation of the estimated probability $\hat{p_{i}}$. The computation of confidence intervals based on this approximation entails, due to the discreteness and skewness of the binomial distribution, severe problems with respect to their coverage probability, even if #*W*_*i*_ and $\#W_{i}\hat{p_{i}}\left(1-\hat{p_{i}}\right)$ are moderate, see [16] for a detailed discussion. Among the various alternatives given by [17] and [16], we employ the Agresti-Coull interval [18]

$$CI\left(p_{i}\right)=\left({\tilde{p}}_{i}-\kappa \sqrt{\frac{\tilde{p_{i}}\left(1-\tilde{p_{i}}\right)}{\left(\#W_{i}+\kappa^{2}\right)}},{\tilde{p}}_{i}+\kappa \sqrt{\frac{\tilde{p_{i}}\left(1-\tilde{p_{i}}\right)}{\left(\#W_{i}+\kappa^{2}\right)}}\right),$$ (4)

which is centered at ${\tilde{p}}_{i}=\frac{\sum_{j\in W_{i}}Y_{j}+\kappa^{2}/2}{\#W_{i}+\kappa^{2}}$ for single-subject learning curves estimated by using the constant model. The interval can be viewed as a standard interval obtained from a sample with *κ*^2^/2 failures and successes added.

If the success probability varies significantly across the trials within window *W*_*i*_, the constant model is no longer the appropriate model to use, since the resulting estimate will be biased (see Results). This bias can be severe, for example if the window *W*_*i*_ is not centered at trial *i* (border effect), i.e. when *t*_*i*_ is close to *t*_1_ or *t*_*n*_, or if the success probability is strongly and rapidly changing within *W*_*i*_.

In situations when the success probabilities within the window are time dependent, the constant model can, for each window *W*_*i*_, be replaced by a generalized linear model (GLM) [13] that allows the logit transform of the success probability to vary linearly over trials. The GLM relates, for trials *j* within a fixed window *W*_*i*_, the success probability *p*_*j*_ to a linear predictor

$$\eta_{j}\left(\beta \right)=\beta_{0}+\beta_{1}\left(t_{j}-t_{i}\right)$$ (5)

with local trial parameters *β* = (*β*_0_, *β*_1_), and *p*_*j*_ = *μ*(*η*_*j*_(*β*)). The inverse relation *η_j_*(*β*) = *μ*^−1^(*p_j_*) = *g*(*p_j_*) is determined by the link function *g*(). Its canonical choice for the binomial family is the logit link $g\left(p\right)=\log(\frac{p}{\left(1-p\right)})$ which provides $\mu \left(\eta \right)=\frac{\exp\eta }{1+\exp\eta }$.

Local parameters *β* are estimated from the trial outcomes within window *W*_*i*_ as

$$\hat{\beta }={\mathrm{argmax}}_{\beta }\sum_{j\in W_{i}}Y_{j}\log\left(\mu \left(\eta_{j}\left(\beta \right)\right)\right)+\left(1-Y_{j}\right)\log\left(1-\mu \left(\eta_{j}\left(\beta \right)\right)\right).$$ (6)

Estimates of the success probabilities are derived as

$${\hat{p}}_{i}=\mu \left(\eta_{i}\left(\hat{\beta }\right)\right)=\mu \left({\hat{\beta }}_{0}\right).$$ (7)

Point-wise confidence intervals for ${\hat{p}}_{i}$ are obtained in the GLM assuming asymptotic normality of ${\hat{\eta }}_{i}$ as

$$\left(\mu \left(\eta_{i}-\kappa s_{i}\right),\mu \left(\eta_{i}+\kappa s_{i}\right)\right),$$ (8)

where *s*_*i*_ denotes the standard deviation of the linear predictor $\eta_{i}\left(\hat{\beta }\right)$ and *κ* is the (1 − *α*/2)-quantile of the standard Gaussian distribution. Standard deviations *s*_*i*_ were determined from the asymptotic covariance matrix of the parameter estimate.

In the generalized linear model with logistic link (logistic regression), estimates are biased if the sample size is small, or counts for a possible outcome are either very high or very low. To account for this problem we used Firth’s bias adjusted estimates [14, 19] implemented in the package logistf for R [20]. In contrast to the standard maximum likelihood procedure used in standard GLM analyses, Firth’s approach maximizes a penalized likelihood function and guarantees finite parameter estimates. All analyses were carried out in R [21].

#### Bandwidth selection of the moving window

Properties of the estimates strongly depend on the size of the analysis window, i.e. on the chosen bandwidth *h* (Eq (1)). Large bandwidths lead to a potential bias of the estimated probabilities, while estimates employing small bandwidths suffer from high variability (see Results). As a criterion for bandwidth selection, we suggest to use the expected mean squared error *(MSEP)* of prediction given by

$$MSEP\left(h\right)=E_{Y}{\left(Y-\hat{p}\left(h\right)\right)}^{2}.$$ (9)

An estimate with minimal *MSEP* balances bias and variability of the estimates. The *MSEP* can be estimated by leave-one-out cross-validation as

$$\hat{MSEP}\left(h\right)=\sum_{i}{\left(Y_{i}-{\hat{p}}_{i}^{\left(-i\right)}\left(h\right)\right)}^{2},$$ (10)

where ${\hat{p}}_{i}^{\left(-i\right)}\left(h\right)$ is the estimate of *p*_*i*_ employing a window *W*_*i*_(*h*)/{*i*}, i.e. with the observation from trial *i* removed.

#### Grand mean learning curves

The calculation of grand mean learning curves for Bernoulli trials was based on a GLM, employing Firth’s bias adjustmend [14, 19] in a fixed effects model centered at time point *i*:

$$\eta_{ji}^{\left(k\right)}=\beta_{0,i}+\beta_{1,i}\left(t_{j}-t_{i}\right)+\beta_{0,i}^{\left(k\right)}+\beta_{1,i}^{\left(k\right)}\left(t_{j}-t_{i}\right)\;$$ (11)

$$\text{with}\;\sum_{k}\beta_{0,i}^{\left(k\right)}=\sum_{k}\beta_{1,i}^{\left(k\right)}=0$$

The mean success probability at time point *i* is given as *β*_0,*i*_, and *β*_1,*i*_ defines the slope of the tangential approximation to the logit transform of the grand mean learning curve. Parameters $\beta_{0,i}^{\left(k\right)}$ and $\beta_{1,i}^{\left(k\right)}$ characterise, under logit transform, the deviation of the learning curve for subject *k* from the grand mean at time point *i*. We use fixed effects rather than random effects since the rodent population is heterogeneous with respect to its learning behavior, as indicated by the estimated individual learning curves (see Results). Whether a subject responds correctly or not depends not only on the learning state but also on other cognitive and non-cognitive factors which can systematically differ among subjects. The fixed-effects model accounts for such inter-individual differences which cannot be modeled properly as random variation in a homogeneous population. Estimated probabilities ${\hat{p}}_{i}^{\left(k\right)}$ for subject *k* at trial *i* can be represented by their logit transform ${\hat{\eta }}_{ii}^{\left(k\right)}=\log\frac{{\hat{p}}_{i}^{\left(k\right)}}{1-{\hat{p}}_{i}^{\left(k\right)}}$. In the GLM framework, standard deviations $s_{i}^{\left(k\right)}$ of ${\hat{\eta }}_{ii}^{\left(k\right)}$ can be obtained from the asymptotic covariance matrix of the parameters $\beta_{.,i}+\beta_{.,i}^{\left(k\right)}$. In the constant model, the variance of ${\hat{\eta }}_{ii}^{\left(k\right)}$ is given by

$$v_{i}^{\left(k\right)}=1/\left(\#W_{i}{\hat{p}}^{\left(k\right)}\left(1-{\hat{p}}^{\left(k\right)}\right)\right).$$

The GLM assumes the logit transforms ${\hat{\eta }}_{ii}^{\left(k\right)}$ to be approximately Gaussian. Values for the grand mean curves are therefore computed as

$$\begin{array}{ll} {\hat{\eta }}_{i}^{GM} & =\left(\frac{1}{\#Subjects}\underset{k\in Subjects}{\sum }({\hat{\eta }}_{ii}^{\left(k\right)})\right)={\hat{\beta }}_{0,i}\\ {\hat{p}}_{i}^{GM} & =\frac{\text{exp}{\hat{\eta }}_{i}^{GM}}{1+\text{exp}{\hat{\eta }}_{i}^{GM}}. \end{array}$$ (12)

with the variance of the logit transform ${\hat{\eta }}_{i}^{GM}$ given by

$$v_{i}^{GM}=\frac{1}{\#Subjects}\sum_{k\in Subjects}v_{i}^{\left(k\right)}.$$

Point-wise confidence intervals for the grand mean are obtained again according to Eq (8) as

$$[\mu \left(\eta_{i}-\kappa \sqrt{v_{i}}\right),\mu \left(\eta_{i}+\kappa \sqrt{v_{i}}\right)].$$ (13)

#### Analysis of continuous behavioral and physiological covariates of learning

In addition to the learning curve itself, we further analysed the temporal dynamics of reaction times for the conditioned responses as an example of a behavioral covariate, and amplitudes of prefrontal cortical potentials evoked by the conditioned stimuli (see Experimental data) as an example of a neurophysiological covariate. As for the GLM in Eq (5), we employed a local linear model [22] using the package lpridge for R [23]. To determine significant changes, point-wise confidence intervals were computed under Gaussian assumptions. Grand means were calculated by population averaging with confidence intervals derived from standard errors.

### Experimental data

#### Animal training and acquisition of behavioral data

We used data from a previous two-way active avoidance experiment in which 20 Mongolian gerbils (*Meriones unguiculatus*) were trained to detect frequency-modulated (FM) sweeps [4]. Animals weighed 80–100 g, and were kept in single cages (24 × 20 × 14 cm^3^) with ambient illumination on a 12:12 h light/dark cycle and free access to food pellets, sun flower seeds, and water. Each animal was handled daily during the experiment minimizing their stress as a prerequisite for the learning task. During handling, animals were monitored to dected signs of pain, inflammation, or other forms of illness. None of the animals showed signs of pain, became ill, or died before the experimental endpoint. After the experiment, animals were deeply anesthetized with pentobarbital (Sigma, Taufkirchen, Germany), and then killed by an intrapulmonary injection of T61 (Hoechst). Animals were treated in accordance with National Institute of Health procedures for care and use of laboratory animals, and experiments were approved by the ethics committee of the state of Sachsen-Anhalt (No. 42502/2-553IfN).

**Fig 1:**
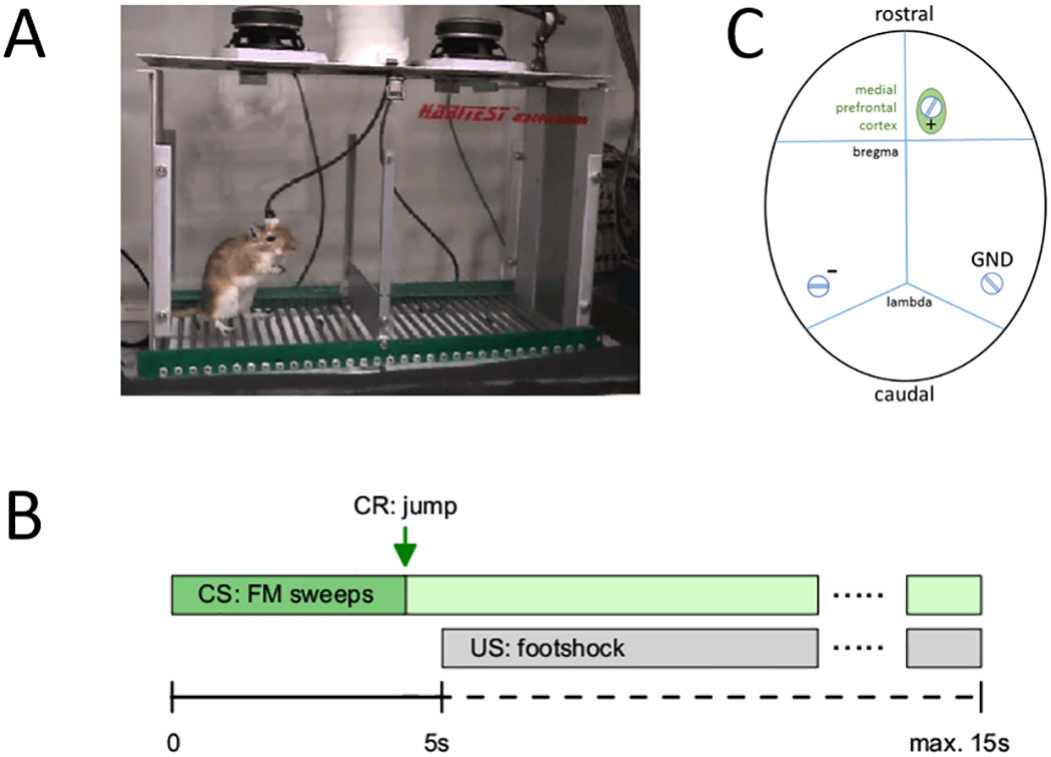
Experimental setup. (A) Snapshot of a gerbil in the shuttle-box. (B) Trial structure of the experiment. (C) Sketch of the setup for the recording of the electrocorticogram. The recording electrode, a stainlees-steel screw, was located in the rostral part of the brain, above the medial prefrontal cortex.

The training took place in a shuttle-box consisting of two compartments separated by a small hurdle (Fig 1A). It was carried out in three consecutive sessions with maximum two sessions per day separated by a pause of at least two hours. The remaining session(s) was performed on the following day. Each session consisted of 60 trials and lasted about 30 minutes. Within each session, and after an initial three-minute phase of habituation to the shuttle-box, a new trial was presented every 30 seconds.

The structure of a trial is shown in Fig 1B. It started with the onset of the conditioned stimulus (CS) which consisted of a sequence of linear FM sweeps presented at an intensity of 60 dB sound-pressure level. The 0.25-s long FM sweeps were repeated with a stimulus-onset interval of 0.5 s for a maximum of 15 s. In each trial, either a sequence of rising (1–2 kHz) or falling (2–1 kHz) FM sweeps was presented as CS. In order to avoid the unconditioned stimulus (US)—a mild electric footshock elicited by 600-*μ*A current pulses delivered through the grid-floor of the shuttle-box—animals had to learn to change the compartment in response to both, rising and falling CSs, by crossing the hurdle within 5 s after CS-onset. If the animal did not change the compartment, the footshock was applied 5 s after CS-onset for a maximum duration of 10 s. A conditioned avoidance response (CR) was defined as a compartment change within 5 s after CS-onset, before US-onset. The CS was immediately turned off after a CR of the animal. An unconditioned response (UCR) consisted of a compartment change in response to the footshock, i.e. an escape response after US-onset. Both CS and US were immediately switched off after an escape response. A response was counted as correct avoidance response, if the animal changed the compartment within the 5-s interval after CS-onset (for further details see [4]).

#### Electrocorticogram recording

Electrocorticograms (ECoGs) were recorded epidurally from the surface of the brain via a stainless-steel skull screw (1.5 mm diameter, Fig 1C) implanted above medial prefrontal cortex (mPFC). For surgery, anesthesia was induced by 400 mg pentobarbital (Sigma, Taufkirchen, Germany) per kg body weight intraperitoneally. All animals resumed normal behavior including feeding 3 hours after surgery, and were given a pause of at least 3 days until the first training. Electrodes were connected to an amplifier via a connector and a cable. Two further parietal skull screws served as reference/ground electrodes. ECoG signals were amplified 10.000 times, low-pass filtered at 100 Hz, and digitized at a sampling rate of 500 Hz (for further details see [4]).

## Results

### Estimation of individual learning curves and their statistical properties

#### Experimental data

**Fig 2:**
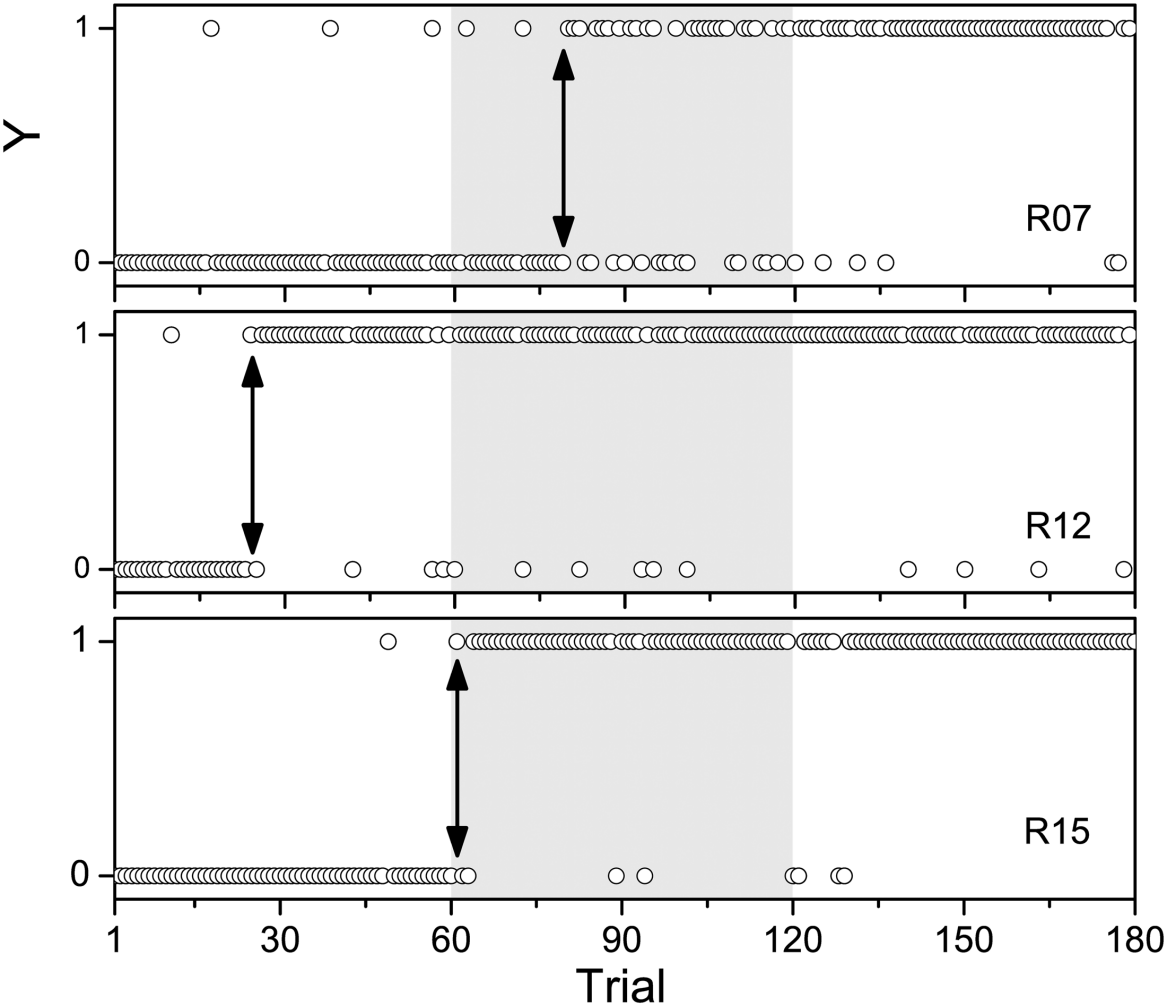
Behavioral responses. Time courses of correct (*Y* = 1) and incorrect (*Y* = 0) responses of three exemplary data sets recorded from gerbils R07, R12, and R15. In total 180 trials were presented in three different sessions of 60 trials each, with a break of at least two hours between two consecutive sessions. Black arrows indicate the approximate transitions from the naive state to the state where the task had been learned by the animal. The three experimental sessions are separated by different background colors.

The quality of learning curve estimation relies on the chosen window size, the employed statistical model, the learning phase, and on discontinuities arising from breaks between training sessions. To systematically explore the influence of these factors, learning curves were estimated from exerimental data obtained from Mongolian gerbils trained with a two-way active avoidance paradigm in the shuttle-box (Fig 1). A correct avoidance response consisted of a compartment change (jump) within 5 s after CS-onset. Fig 2 shows correct (*Y* = 1) and incorrect (*Y* = 0) responses as a function of trial, exemplarily for three rodents (R07, R12, R15). The figure emphasizes the individuality of the learning process for different subjects. For each animal, the learning transition occurs at a distinctive point in time, within the second session (R07), within the first session (R12), or at the border between first and second session (R15).

**Fig 3:**
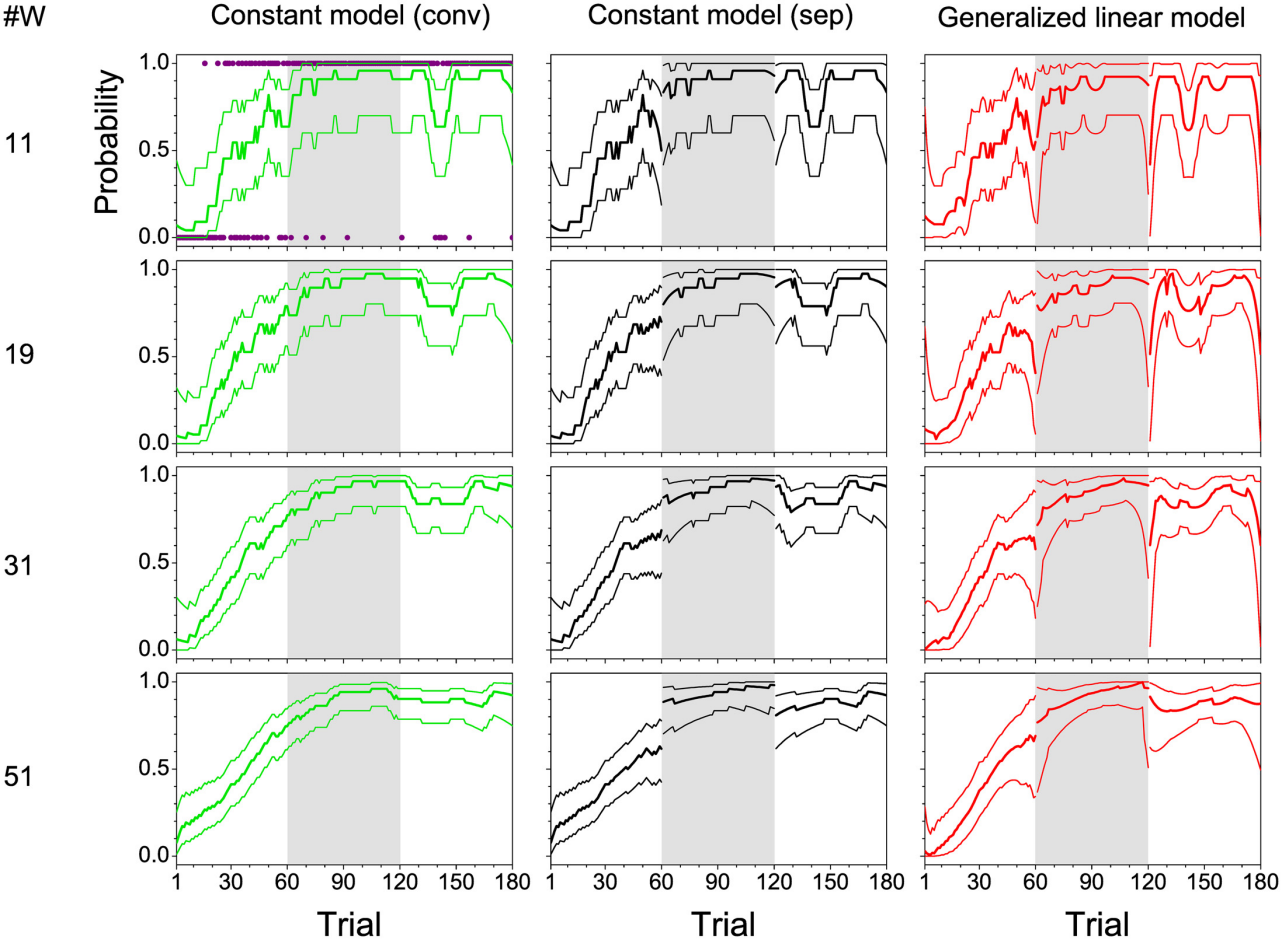
Exemplary learning curves. Point-wise success probabilities (thick lines) and their confidence intervals (thin lines) from a single animal. Estimation was carried out with three different approaches: constant model with moving window analysis across sessions (conv, left column, green lines), constant model with moving window analysis within sessions (sep, middle column, black lines), and generalized linear model with moving window analysis within sessions (right column, red lines). Results for four different window sizes are shown: 11, 19, 31, and 51. The underlying correct (1) and incorrect (0) single trial responses are displayed by pink symbols in the top left panel.—The three experimental sessions are separated by different background colors.

In Fig 3, examples of learning curves estimated from the correct and incorrect responses (indicated by pink symbols in the upper left panel of Fig 3), are shown for a single rodent for four different window sizes (11, 19, 31, 51) and for two different statistical models (constant model and GLM). Results from a conventional moving window analysis (constant model (conv)), in which the window is moved trial by trial across all sessions irrespective of session breaks, are displayed in the panels of the left column of Fig 3. The panels in the middle column of this figure show findings from a moving window analysis likewise based on the constant model but now applied separately to each session (constant model (sep)). Here, approximate point-wise confidence intervals were computed according to the Agresti-Coull interval (Eq (4)), which avoids the problems of asymptotic normal approximation of binomial proportion intervals. The panels on the right column of Fig 3 show the session-wise learning-curve estimates obtained with the GLM using Firth’s bias-adjusted estimates.

Whereas the moving window analysis across session borders yielded smooth, continuous learning curves (constant model (conv), left column of Fig 3), step-like increases and decreases (discontinuities) between sessions appeared, when moving window analysis was carried out separately for each session (constant model (sep), middle column, and GLM, right column of Fig 3). In contrast to the constant model (sep), the session-wise GLM revealed fast changes of success probabilities at the session borders within less than 10 trials, for example steep increases at the beginning of sessions 2 and 3 (right column of Fig 3, particularly for small window sizes). These changes can emphasize or de-emphasize the discontinuities in learning performance between the last trial of a session and the first trial of the following session, and affect session-wise analysis of learning.

In all three approaches, the larger the window size, the smoother the learning curves and the smaller the width of the confidence intervals. This finding reflects the decreasing variability of the estimate with increasing sample size within the window. Moreover, the slope of the learning curves decreased with increasing window size in all three approaches. However, compared to the learning curves estimated by means of the constant model (conv) and constant model (sep), the slopes obtained with the GLM remained steeper at all window sizes. To obtain a more quantitative assertion of this observation, slopes of single-subject learning curves were calculated within sessions as differences in performance between consecutive trials, separately for the four window sizes, and for constant model (sep) and GLM. Maximum slopes were averaged across subjects. Although these average maximum slopes decreased with increasing window size for the two models, they always remained nearly twice as large with the GLM compared to the constant model (sep) for each window size. For example, for the GLM, the average maximum slope of 0.172 ± 0.005 per trial was obtained for window size 11, and of 0.044 ± 0.004 per trial for window size 51. For the constant model (sep), the average maximum slope was only 0.089 ± 0.001 per trial, and 0.021 ± 0.001 per trial, respectively.

#### Simulations

Variation of confidence intervals and slopes indicate, that learning curve estimation suffers from random and systematic errors depending on window size and statistical model. Random errors can be assessed from the experimental data by means of variance and confidence intervals. However, the evaluation of the systematic errors requires the knowledge of the true success probabilities underlying the observed responses. Systematic errors can be assessed by estimation bias which is the difference between the expected estimated value and the true value. They can also be revealed by the coverage probability which is the probability that the confidence interval contains the true value. If the coverage probability is significantly below its nominal 95%-value, confidence intervals are systematically undersized or misplaced.

To assess systematic errors, we carried out a simulation study with known true success probabilities. We estimated a population learning curve from our data by a GLM fixed effects model, and used its sucess probabilities to randomly generate 1000 different trial sequences of 180 correct or incorrect responses closely resembling our experimental data. For each of the simulated trial sequences, we estimated a learning curve using the constant model (Eq (2), conv and sep), and the GLM (Eq (7)). In the conventional analysis (conv), windows were moved across session borders, whereas the other analyses (sep and GLM) were carried out separately for the three sessions (trials 1–60, 61–120, and 121–180). Computations were performed for the same four window sizes (11, 19, 31, 51) as in Fig 3. We then assessed the effect of session breaks (conventional analysis versus session-wise analyses), window size, and model (constant model versus GLM) on different measures of random and systematic estimation errors.

**Fig 4:**
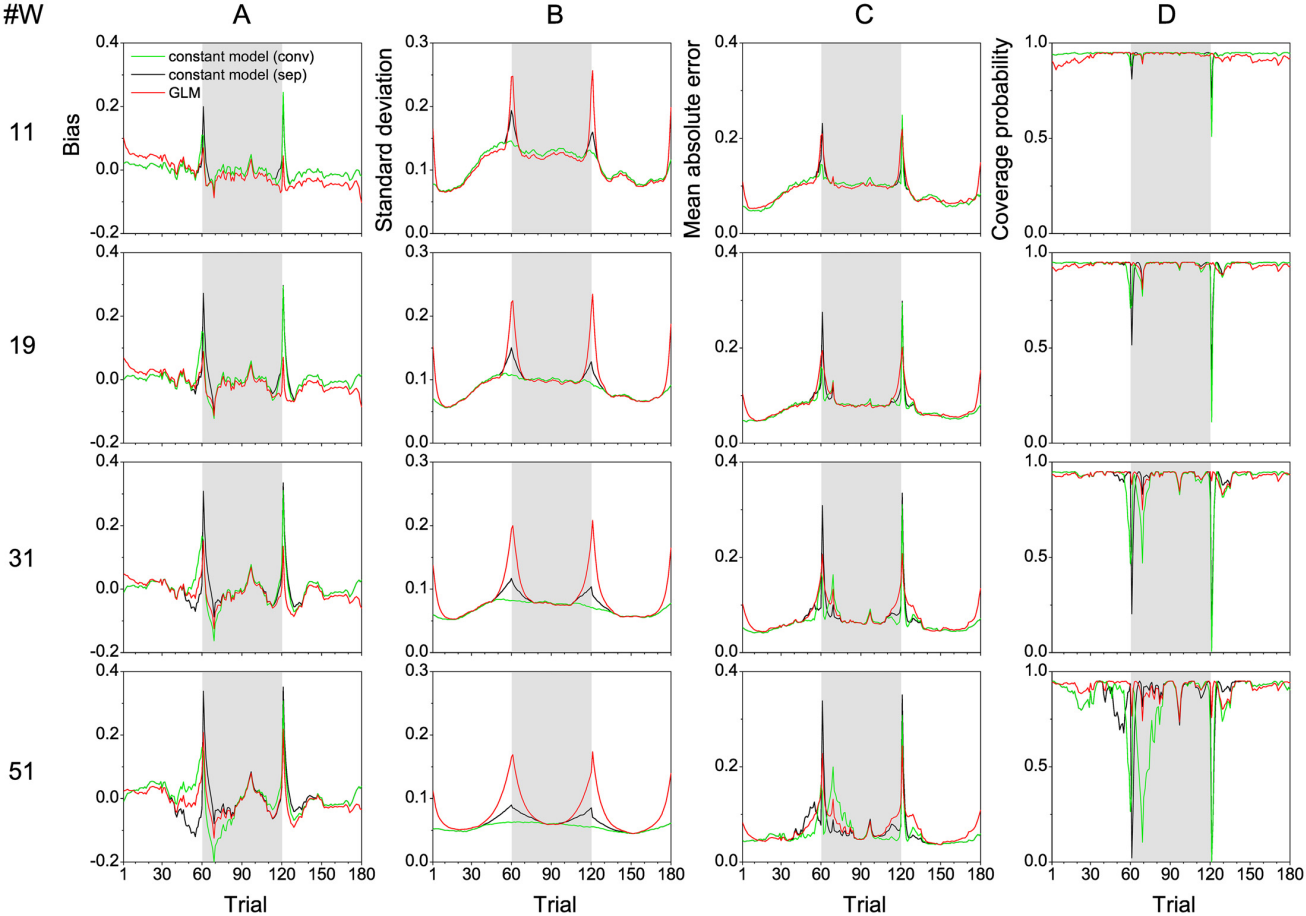
Simulation. Results were derived from 1000 simulated response sequences (180 trials) based on a population learning curve (n = 20) estimated from the data using a GLM and a window size of 15. Individual learning curves (n = 1000) were estimated from these data for four different window sizes (11, 19, 31, and 51) employing either a constant model in conventional moving window analysis across sessions (conv, green lines), a constant model within sessions (sep, black lines), or the GLM within sessions (red lines). (A) Bias, i.e. the mean difference between original learning curve and curves estimated from each of the simulated response sequences. (B) Standard deviation (SD), (C) mean absolute error (*MAE*), and (D) coverage probability of confidence intervals of the estimated learning curves. Note the large values of all measures as well as the strong differences between the three approaches at the session borders.—The three experimental sessions are separated by different background colors.

Results of the simulation are summarized in Fig 4. In all panels of this figure, green curves result from conventional analysis (conv), black curves from session-wise analysis with the constant model (sep), and red curves from session-wise analysis with the GLM. The panels of column (A) show the trial dependence of the bias, i.e. the differences between the grand mean across the 1000 simulated trial sequences of estimated success probabilities and the true population learning curve used to simulate the trial sequences. Positive bias indicates overestimation and negative bias underestimation of success probability. Generally, the magnitude of bias increased with window size. Particularly large positive and negative bias even for small windows was found in the first five trials of sessions two and three. In these phases, a fast increase was seen in many individual learning curves. Bias observed with GLM was generally lower than with the constant model (both conv and sep).

Panels in column (B) of Fig 4 show for different window sizes, the standard deviation (SD) of the estimates as a measure of random errors, trial by trial. As expected, we observed an overall decrease of SD with increasing window size. The SDs of the three approaches did not differ much from each other within the sessions. Compared to the conventional analyses, however, SD strongly increased in the session-wise analysis towards the session borders where the effective window size decreased. This increase was most pronounced for the GLM.

The mean absolute error, $MAE\left(i\right)=\sum_{s=1}^{1000}\mathrm{abs}\left({\hat{p}}_{i}^{s}-p_{i}\right)$, for trial *i* is displayed in column (C) of Fig 4. *MAE* quantifies the combined effects of bias and variance and is a robust measure of the quality of the estimate. It shows a dominant effect of variability for smaller window sizes and a decrease of overall estimation quality especially at the session borders.

Panels in column (D) of Fig 4 display the trial dependence of the coverage probability of the nominal 95%-confidence interval for different window sizes. Empirical coverage probability should be itself close to 95%, otherwise estimated confidence intervals are undersized or misplaced. Deviations of coverage probability from its 95% nominal value therefore indicate systematic errors. Similar to bias, deviation of coverage probability from 95% increased with increasing window size. Coverage probabilities much smaller than 95% were observed for the conventional analysis at the session borders, and for larger window sizes also within the session. Low coverage probabilities were also found for the session-wise application of the constant model (sep), at the beginning of sessions 2 and 3, particularly at trials 61 and 121, i.e. when bias had its maximum. Therefore, in trials with low coverage probability, the interpretation of learning curves obtained with the conventional analysis, or with the session-wise constant model becomes highly questionable. As with bias, deviations were much more pronounced for the constant model (conv), and for the constant model (sep) than for the GLM. Thus, systematic errors were lowest for the GLM, but only when applied in a moving window analysis stopping at the session borders. Similar to conventional analysis, large systematic errors were found with the GLM when analysis windows were moved across session borders (see S1 Fig). Just as the constant model (sep), the GLM is unable to model abrupt, step-like changes of performance across session breaks.

### Selection of window size

**Fig 5:**
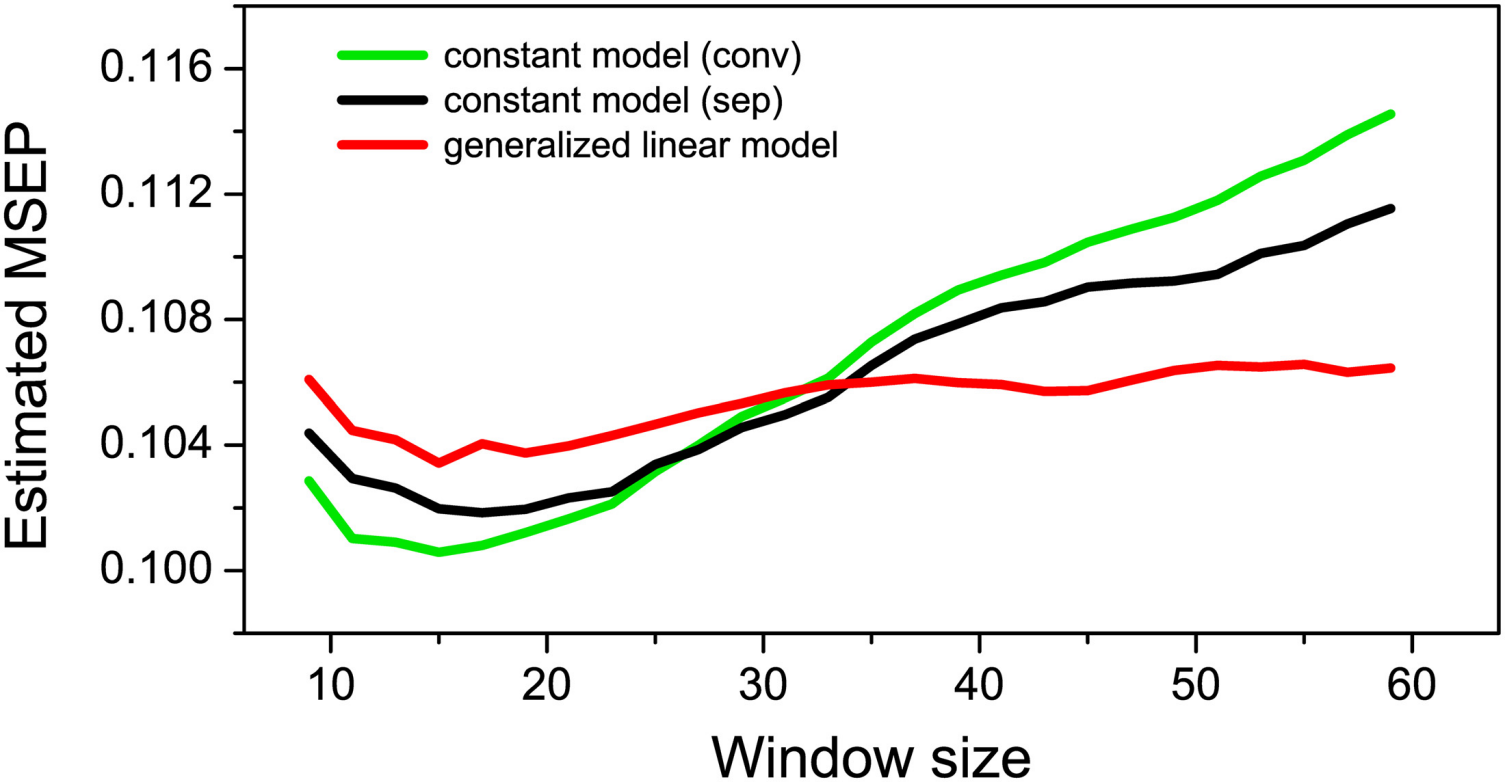
Cross-validation of window size. Window-size dependence of the estimated mean squared error of prediction (*MSEP*) determined across trials and subjects in a leave-one-out cross-validation procedure. Results are shown for the conventional analysis, i.e. the application of the constant model in a moving window analysis across session breaks (conv, green line), for the application of the constant model (sep, black line), and for the GLM (red line) in a moving-window analysis separately for each session.

Our simulation shows how random and systematic errors of learning curve estimation strongly depend on window size. Window sizes therefore should be chosen with the aim of minimizing both random and systematic errors. However, systematic errors assessed by measures of bias and coverage probability as in the simulation analysis cannot be determined from the experimental data for which the true success probabilities underlying the observed responses are not known. Though, a combined measure of estimation errors can be calculated from the experimental data through estimates of the expected mean squared error of prediction (Eq (9)) derived from a cross-validation procedure (Eq (10), *MSEP*). By means of *MSEP*, we quantified how well the observed response in each single trial could be predicted by the estimated success probability as a function of window size. Fig 5 shows the window-size dependence of the estimated *MSEP* for conventional analysis (conv, green trace), the session-wise constant model (sep, black trace), and the session-wise GLM (red trace). *MSEP* is affected by an increase in random errors with shorter, as well as an increase in systematic errors with longer trial windows. To minimize and balance the influence of random and systematic errors, the use of a window size is recommended where *MSEP* displays a minimum. For all three methods, the smallest *MSEP* values are found for window sizes in the interval between 13 and 19. Notably, the estimated *MSEP* itself is a function of the observed data and therefore a realisation of a random variate. In the window-size interval between 13 and 19, differences between the *MSEP* values of each individual *MSEP* curve are small, below about 1% of the overall variability. Therefore, any of these window sizes might respresent the minimum *MSEP*. For further analysis we chose the largest of these window sizes, viz. 19, because the variance of the estimation decreases with increasing window size (see Fig 4B). For window sizes larger than 33 trials, the estimated *MSEP* computed for the GLM was smaller than that for the constant model (sep). This is consistent with the finding from our simulation that the use of the GLM reduced systematic errors of learning curve estimation even for larger window sizes.

### Intra- and intersubject variability of learning curves

**Fig 6:**
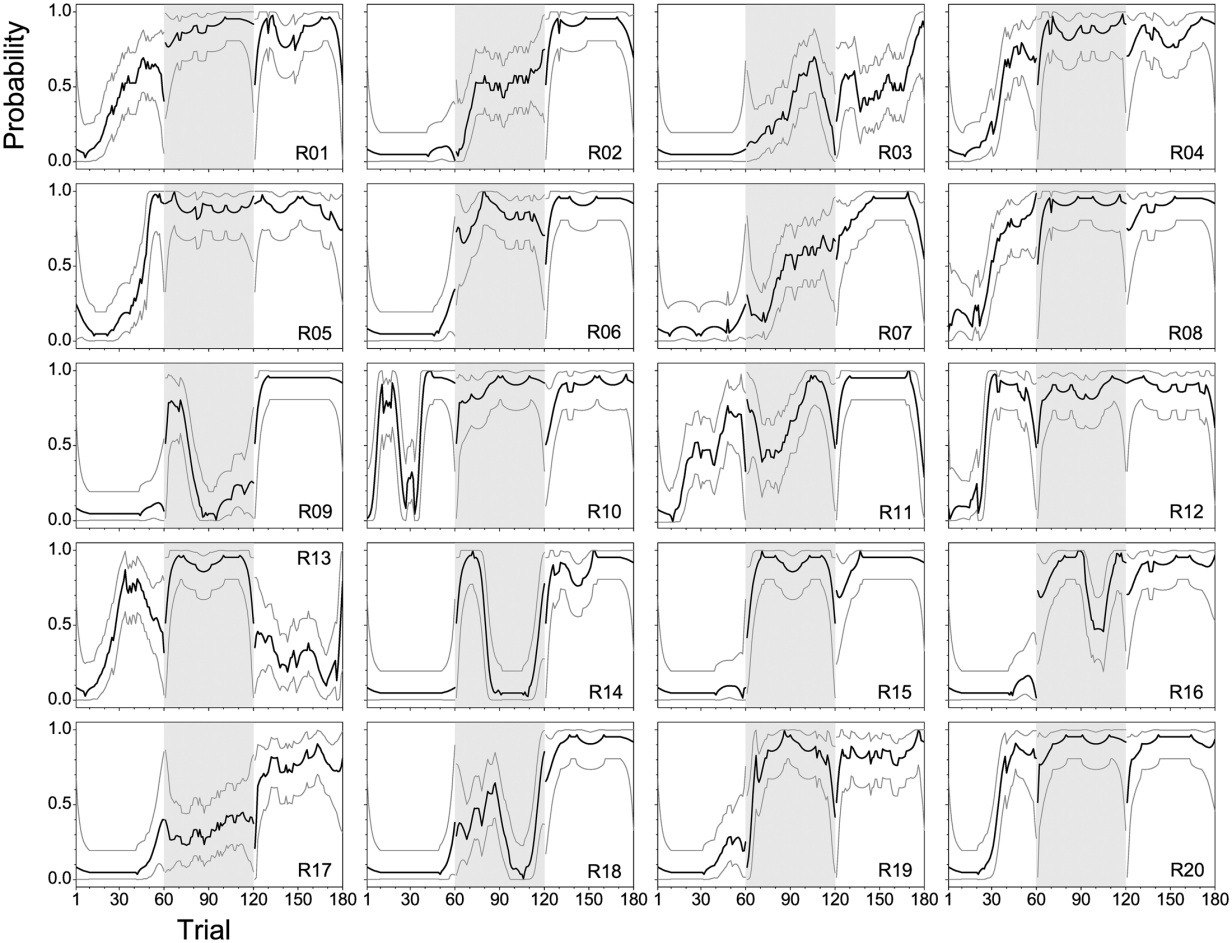
Individual learning curves estimated with a GLM. Learning curves (black lines) for the 20 individual rodents (R01 to R20) with 95%-confidence intervals (thin grey lines) derived from the constant term of a GLM maximum likelihood estimation with a moving window of 19 trials.—The three experimental sessions are separated by different background colors.

Learning curves display a large variability between subjects. Fig 6 shows learning curves of all 20 rodents together with their 95%-confidence intervals estimated for each subject individually with the GLM and a window size of 19 trials (see above). Non-overlapping confidence intervals can be used to identify non-random changes of performance between consecutive trials or within a narrow range of neighboring trials as an indicator of learning. The prevailing pattern observed in a large number of animals (*n* = 10; R01, R04, R05, R08, R10–R13, R17, R20) was an initial increase of the success probability during the first session. For all other animals, the first non-random increase in task performance occurred in the second session. The transition between the different learning states occurred mainly within a rather narrow range of about 10 to 20 trials, but could also appear even more rapidly, almost from one trial to the next. After an initial increase in performance, some animals showed a transient decline in performance in the course of a session (e.g. R03, R09, R10, R11, R13, R14, R16, R18), and/or rapid increases in learning performance within the first 10 trials of session 2 or 3 (e.g. R01, R04, R08, R09, R10, R14, R20). For comparison, single-subject learning curves derived from conventional analysis and from session-wise analysis based on a constant model are shown in S2 and S3 Figs, respectively. As will be demonstrated by the population analysis below, moving window analysis across session borders, and the application of a constant model blur the underlying temporal dynamics of learning.

### Population learning curve and its behavioral and physiological covariates

**Fig 7:**
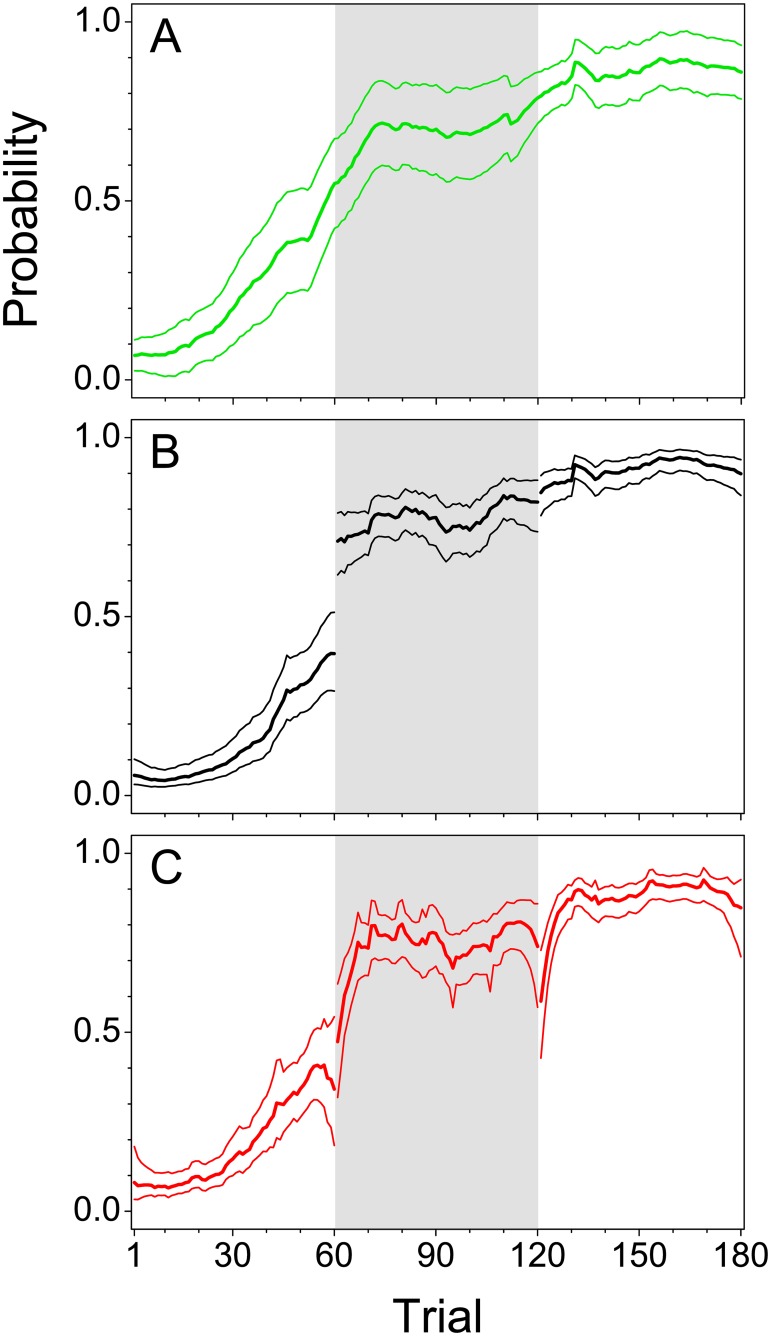
Population learning peformance. (A) Grand mean learning curve (*n* = 20, thick green line) obtained by conventional analysis employing the constant model (conv) in which the window is continously moved across session borders. Point-wise 95% confidence intervals (thin green lines) are estimated by means of standard errors. (B) Population learning curve (*n* = 20, thick black line) estimated by the constant model (sep). Point-wise 95% confidence intervals (thin black lines) were approximated by the Agresti-Coull interval. (C) Population learning curve (*n* = 20, thick red line) estimated by a generalized linear fixed effects model. Point-wise 95% confidence intervals (thin red lines) were obtained from Firth’s penalized likelihood approach for the GLM.—In all three cases, the window size was 19 trials. The three experimental sessions are separated by different background colors.

Fig 7 shows populations learning curves and their confidence intervals derived from the responses of 20 subjects with the three different statistical approaches introduced in Fig 3. Fig 7A displays the conventional arithmetic grand mean learning curve obtained by a continuous moving window analysis across session borders (constant model (conv)). Confidence intervals were based on the standard errors of the mean. This effectively assumes a random effects model with, for fixed trial number, a Gaussian distribution of individual success probabilities and approximately Gaussian distributions for their individual estimates; note, however, that especially the first assumption is violated (see Fig 6). The analysis resulted in a smooth, sigmoid population learning curve, without any discontinuities at the session borders and therefore masks essential effects caused by the session breaks.

Fig 7B and 7C show population learning curves and their confidence intervals estimated separately for each of the three sessions using a constant (B) and a generalized linear (C) fixed effects model described by Eq (11), respectively (see Materials and Methods). Confidence intervals of learning curves estimated by a fixed effects model were smaller than those obtained from standard errors of the mean (see Fig 7A). Fixed effects models provided not only statistically more appropriate, but also more precise estimates than arithmetic averaging. The constant fixed-effects model is known to suffer from bias at session borders, due to asymmetric windows, if the learning curve is steep (see also Fig 4). This bias is avoided in case of the GLM, at the cost of increased variability compared to the constant model.

Similar to the conventional analysis, learning curves obtained from the fixed effects models showed a moderate increase of performance in the second half of the first session, although this increase was smaller and occurred somewhat later compared to the conventional model. With the constant fixed effects model (Fig 7B), a step-like increase from the last trial of session 1 to the first trial of session 2 appeared in the estimate. This step may reflect an improvement of performance during the session break without training, e.g. by memory consolidation. In the conventional analysis (Fig 7A), such discontinuities in performance were obscured. Population learning curves based on the GLM, however, additionally revealed fast and strong increases of performance within the first five to ten trials of sessions 2 and 3, respectively (Fig 7C). Opposite to the constant fixed effects model, performance in the first trials of session 2 did not significantly differ from the last trials of session 1, or was even worse in the first trials of session 3 than in the last trials of session 2. After the rapid increase at the beginning of session 2 and 3, the performance level reached a plateau in less than 10 trials, and exceeded that of the preceding session. In the case of the generalized linear fixed effects model, the increase in performance from session 1 to session 2 would not occur unobserved as in the conventional approach, but eveals a fast learning process at the beginning of session 2.

**Fig 8:**
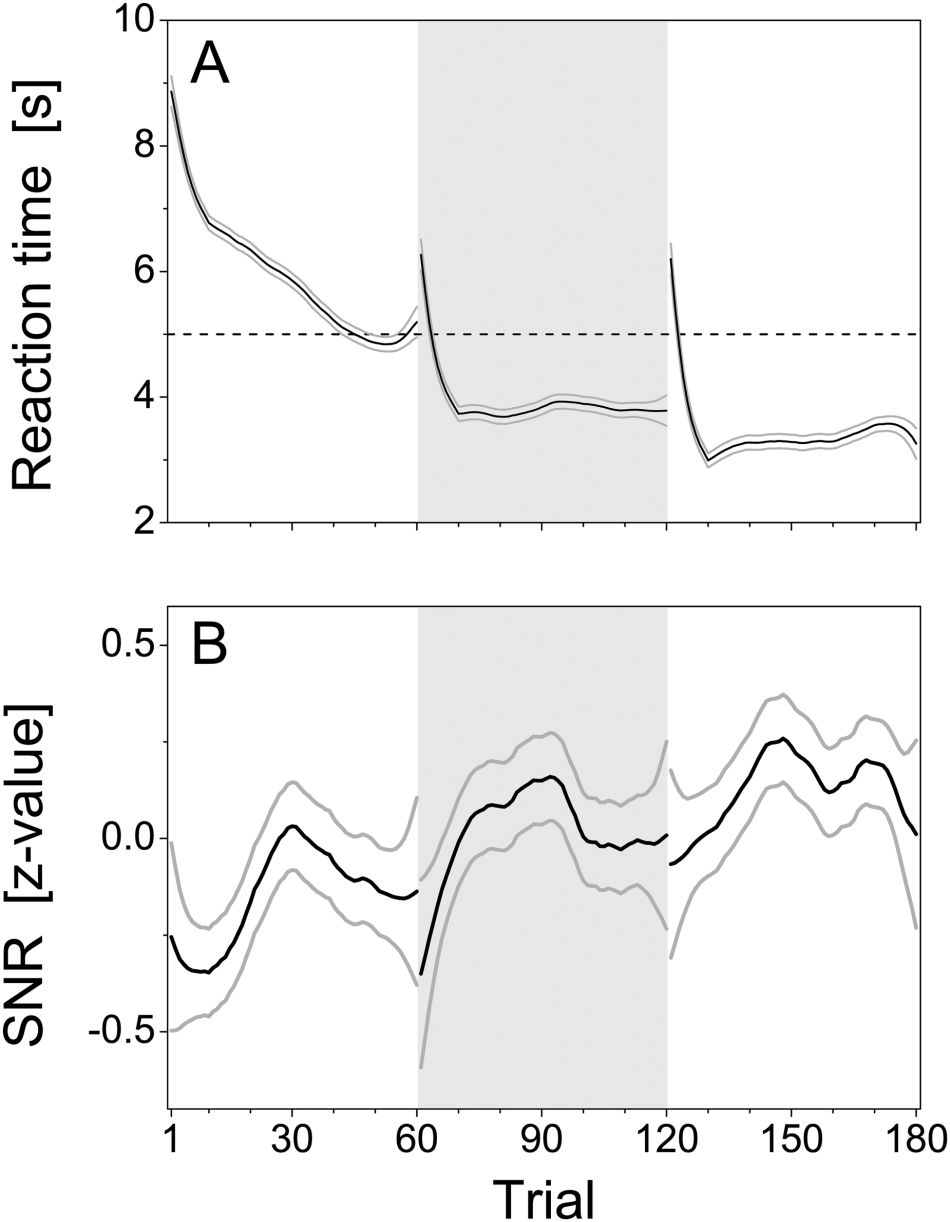
Covariates of learning. Results obtained from behavioral and physiological covariates. (A) Grand mean of reaction times (*n* = 20, black line) obtained by local linear smoothing over trials with window size 19. Point-wise 95% confidence intervals (grey lines) were determined from standard errors under Gaussian assumptions. The 5-s threshold for a correct response is indicated by the dashed horizontal line. (B) Grand mean of the z-standardized signal-to-noise ratio of the CS-evoked electric potential at the prefrontal electrode over trials after local linear smoothing with window size 19 (*n* = 20, black line). Point-wise 95% confidence intervals (grey lines) were determined from standard errors under Gaussian assumptions.—In both panels, the three experimental sessions are separated by different background colors.

Further insight into the learning process was obtained from behavioral and physiological covariates recorded during training. Fig 8A shows the population reaction time curve and its confidence interval as a function of trial obtained by local linear smoothing (see Materials and Methods). Even before the initial increase of the learning curve in the first session (see Fig 7), the reaction time already decayed in a fast initial phase by about 2 s within the first 10 trials. A slower decrease by about two further seconds followed within the subsequent 40 trials. Note that learning performance did not increase until the second half of the slow phase of the reaction time decay (see Fig 7).

The fast drop in reaction time at the beginning of sessions 2 and 3 mirrors the initial increase the population learning curve in these sessions revealed by the use of a GLM (Fig 7C). The reaction time was larger than 5 s at the beginning of these two sessions, but only for a few trials. Then it quickly dropped below the 5-s threshold, reaching a plateau for the remaining session. This initial drop of the reaction time became steeper with each consecutive session, and the plateau decreased in steps across sessions. This mirrors the step-like increase in performance level across sessions seen in population learning curves estimated with the fixed effects models (Fig 7B and 7C).

The behavioral changes displayed by learning performance and reaction time were also reflected by neurophysiological changes. During the training, CS-evoked cortical potentials were obtained from ECoG recordings over prefrontal cortex as a neurophysiological covariate of learning. In each trial, the magnitude of this CS-evoked potential was quantified by its signal-to-noise ratio (SNR) relative to pre-stimulus baseline. SNR values were calculated for each trial as the ratio of the logarithm of the root-mean-square (RMS) amplitude of the ECoG from 0 to 0.5 s after and during a 0.5-s long baseline before CS-onset. For each subject, SNR values were z-standardized across trials. Fig 8B shows the population SNR curve along with its confidence interval obtained by local linear smoothing. The SNR varied within and across sessions. SNR increased at the beginning of each session, reached a maximum after about 30 trials, and then decayed again. In the first session, SNR increased to its session maximum during the slow phase of reaction-time decay, just before the initial increase of the population learning curves. Also, SNR showed a steep increase during the first 10 trials of session 2, which paralleled the fast decay of reaction time and the fast increase in performance observed in the population learning curves estimated with a GLM. Besides within-session variation of SNR, the mean SNR within a session increased from session to session, which might be related to the step-like changes of reaction time and performance levels across sessions. Apparently, changes in prefrontal, CS-evoked cortical potential within and across sessions were highly correlated with reaction time and behavioral performance.

## Discussion

The aim of the present work was to improve the precision and the accuracy of the analysis of learning curves, and to allow for a characterization of the learning dynamics at high temporal resolution, trial-by-trial within a single session, as well as across sessions. To achieve this—both for single subjects and on the population level—we introduced appropriate statistical methods for estimating Bernoulli success probabilities and their confidence intervals in small trial windows. Properly determined confidence intervals yield criteria for non-random changes in performance in relation to learning, which are less prone to errors than often used ad hoc criteria (e.g. three consecutive correct responses), predefined thresholds of the success probability (e.g. 0.5), or statistical tests based on Gaussian assumptions [5].

Using established statistical methods, we aimed to minimize the assumptions about the learning process itself, still staying as close as possible to the most frequently used, conventional moving window analysis. An approach for the trial-by-trial analysis of learning curves which has some similarity with this work has been developed by [24]. This highly sophisticated approach is based on the estimation of a state-space random-effects model of the learning process. Although this approach is only based on model assumptions that are very general and apply to most behavioral data, it is more complex and deviates from conventional learning curve analysis since it explicitly models the underlying learning process. Other trial-by-trial analyses of learning are based on strong assumptions about the learning process itself, as it is, for example, the case with reinforcement-learning models. Although these models can explain several aspects of avoidance learning [25–27], they have been rarely applied to experimental data, because it is difficult to obtain stable parameter estimates from them.

### Reducing errors in the estimation of learning curves by appropriate statistical methods

Our simulations showed that learning curve estimation suffers from systematic errors. Particularly, estimations based on the constant model were severly biased in numerous trials. Moreover, estimated confidence intervals in the same trials were largely undersized or misplaced, as revealed by the coverage probability being significantly below its nominal 95%-value. Such systematic errors were found during phases of large and fast performance changes, e.g. at the beginning of sessions 2 and/or 3. These estimation errors led to a decrease of the slope of single-subject and population learning curves, and obscured fast, learning-related changes in responding [9]. Notably, with experimentally obtained learning curves, systematic errors might be even larger. Compared to the population learning curve used in the simulation, individual learning curves are expected to be less smooth and success probabilities therefore less constant. Similar, systematic errors occurred when windows were moved across session breaks obscuring fast performance changes at the session start as well as step-like changes in performance levels across sessions.

Systematic errors arise from varying success probabilities within a trial window. As changes in success probability over trials are a hallmark of learning, a constant model will never be able to describe the learning process exactly. To account for these changes, we introduced a GLM, and employed Firth’s penalized likelihood estimation suitable for small window sizes. In accordance with the reduction of systematic errors in our simulation, analysis of our experimental data with a GLM yielded steeper learning curve slopes and revealed fast and strong increases in population performance within the first five trials of these sessions, which were not detected by the constant model. Corresponding fast and strong behavioral and physiological changes were found in the continuous reaction times and the magnitude of evoked prefrontal cortical potentials, respectively. Moreover, similar changes in responding at the beginning of post-acquisition sessions have been repeatedly reported in avoidance learning, a phenomenon called ‘warm up’. All these findings strongly suggest that the fast changes detected by the GLM approach were true changes, although confidence intervals at session borders were larger for the GLM than for the constant model.

Both the observed random and systematic errors were not only dependent on the chosen model, but also on window size and learning phase. Systematic errors (bias) generally increased with increasing window size, whereas random errors of the estimation assessed by variance and confidence intervals decreased. Smaller random errors can be explained by the increase of sample size with window size.

Systematic errors did not increase for all trials, but only during phases of fast and large performance change. The magnitude of systematic errors apparently depends on the temporal scales of the involved learning processes in relation the chosen window size. If changes within a given window are strong and relatively fast in comparison to window size, the proportion of trials in the window that differ in their success probability from the estimated trial will increase. Also, with growing window size, estimation becomes increasingly influenced by distant trials from a different phase of the learning process potentially governed by different success probabilities. Thus, window size is a major determinant of the quality of learning curve analysis. By cross-validation and minimization of *MSEP*, which is an overall measure of estimation error, we determined with all three approaches optimal window sizes of 13 to 19 trials for our data. These numbers fit well to the observed temporal scales of performance changes which were on the order 5 to 25 trials.

Employing a GLM seemed to be advantageous for protecting estimation against systematic errors, particularly when the chosen window size was large relative to the temporal scale of the learning-related performance changes. However, systematic errors still increased with increasing window size, although to a lesser extent. Presumably, changes of the success probability within the window became non-monotonic, violating the assumption of the GLM. In contrast to the systematic errors, however, random errors of the GLM were larger than of the constant model (sep). This can be explained by the increased number of parameters that have to be estimated in the GLM, which increases the degrees of freedom, and therefore decreases the precision of the estimates. Hence, if the length of the window is at or below the expected temporal scale of learning, the choice of a constant model can be appropriate as well, and would then yield a higher precision.

### Implications for the analysis and interpretation of experimental data

To reduce systematic errors in estimating learning curves from our experimental data, we combined the following methodological steps: (1) the use of a GLM and a fixed effects model fitted by Firth logistic regression for single subject and population analysis, (2) the selection of an optimal window size by cross-validation, and (3) session-wise estimation. The application of these measures yields results differing from conventional analysis, and thus significantly contributes to a proper interpretation of the avoidance learning process.

The major objective of reinforcement learning is maximization of reward and minimization of punishment. In avoidance learning, this is equivalent to a reduction in footshock exposure. The first significant change in behavior and physiology was a fast decay in reaction time during the first 10 trials of the first session (Fig 8A). Although this change was not accompanied by a change in avoidance performance, a decay in reaction time shortens the exposure to the footshock, which was turned off by an escape response. Therefore, this decay presumably reflects a first phase of instrumental learning to escape from the shock [12]. This phase was followed by a second, slower phase of reaction time decay over about 40 trials, that might reflect a second instrumental escape learning phase, in which animals acquire the temporal relationship between CS and footshock (US) onset. The simultaneous increase of the magnitude of the CS-evoked prefrontal cortical potential could reflect aspects of forming Pavlovian associations between CS and US known to be involved in shuttle-box avoidance learning [4, 12]. In the second half of this phase, reaction time further decreased reaching the avoidance criterion of 5 s reflected in a first increase in avoidance performance. This raises the question, whether early correct responses at reaction times below 5 s were genuine avoidance responses. In contrast to an avoidance response controlled by the CS predicting the footshock, these responses might be due to a premature timing of an escape response controlled by the US, which is primed but not elicited by the CS. This could explain that animals only reached success rates of below 50% in this early acquisition phase: If animals timed their escape response by the CS-US interval, and if the error distribution of this interval estimation were symmetric, correct responses would occur in maximally 50% of the cases.

Fast, transient increases of performance, decay in reaction time, and increases in the magnitude of evoked prefrontal potentials were observed during the first ten trials in sessions 2 and 3. Similar changes in responding have been described at the beginning of post-acquisition sessions in various avoidance paradigms [8, 28, 29], and have been called ‘warm up’, which is interpreted as a reinstatement of memory, i.e. a fast relearning of instrumental responses weakened by memory interference due to inter-session activities [27]. However, the fast performance increase in session 2 differed from the described ‘warm up’ phenomena, as the learning performance in the first trial did not fall below the performance level reached in the preceding session 1, and the fast performance increase largely exceeded the level reached in session 1.

Changes in performance were not only found within, but also across sessions. Thus, levels of behavioral performance increased from one session to the next, markedly between session 1 and 2. This was also reflected by decreasing levels of reaction time, and increasing session means of the magnticude of CS-evoked potentials across session. These across-session changes might be related to memory consolidation during session breaks, which can improve performance in the time between sessions, especially when animals had the opportunity to sleep [6, 7]. However, changes in performance level did not occur unobserved during the session break, but were instantiated within the first trials of a session. Seemingly, increases in performance levels were learned within a few trials. To become behaviorally effective, consolidation might therefore require fast learning in the succeeding session. Improvement of responding might thereby not only rely on learning new associations, but also on a better utilization of previously learned information. Such a view would extend to the current interpretation of ‘warm-up’ phenomena as reinstatement of memory.

Finally, it should be noted that inter-individual variability of learning was high. Initial increase in avoidance performance occurred at different times in different animals, even not always within the first session. Also, the initial increase tended to be faster in single subjects. Moreover, in learning curves of some subjects, within session drops in performance were observed after a success rate of about 80% had been reached for several trials. Whereas possible fatigue over trials should be similarly observed in all sessions, behavioral and physiological changes towards the session end depended on the learning state, and might therefore reflect a decrement in attention or motivation. Alternatively, these changes might be due to a fall back to an effective escape strategy by responding to the US after having been primed by the CS. Further statistical methods have to be developed that cope with biases arising from trial-by-trial inhomogeneities of variance across subjects [9, 10].

In summary, our results show that even in simple tasks learning can be multi-phasic, and involve multiple behavioral and physiological processes acting on different time scales [1, 12]. Such a dynamic view of learning is in accordance with studies characterizing learning as governed by the interplay of various conflicting and cooperating brain systems [2]. As noted by [8], describing such a rich learning dynamics only on a molar level, e.g. by session means of success rates, might be too gross for an adequate understanding of learning. Otherwise, a molecular description of learning by the responses of each single trial as by reinforcement learning models must also account for the described multi-scale learning dynamics [27]. In any case, a detailed, quantitative assessment of the behavioral learning dynamics, as provided by our approach, is crucial for the proper interpretation of the various behavioral and neural changes occurring at different time scales during a learning task.

## Supporting Information

S1 Fig Random and systematic errors of learning curve estimation with and without accounting for session breaks. Simulations were carried out as in Fig 4. (A) Bias, (B) standard deviation (SD), (C) mean absolute error (*MAE*), and (D) coverage probabilities of confidence intervals of the estimated learning curves for four different window sizes (11, 19, 31, and 51). Green and blue curves represent moving window analyses across session borders employing a constant model (conv) and a GLM, respectively. Red curves show results of a GLM with session-wise moving window analysis where session breaks have been taken into accout. Similar systematic errors (bias and low coverage probabilities) are found for the two moving window analyses not accounting for session breaks.—The three experimental sessions are separated by different background colors.
(TIF)

S2 Fig Individual learning curves based on the constant model (conv). Learning curves (black lines) for the 20 individual rodents (R01 to R20) with 95%-confidence intervals (thin grey lines) derived from a constant model (conv) with a window of 19 trials moving across trials and session borders.—The three experimental sessions are separated by different background colors.
(TIF)

S3 Fig Individual learning curves based on the constant model (sep). Learning curves (black lines) for the 20 individual rodents (R01 to R20) with 95%-confidence intervals (thin grey lines) derived from a constant model employed in a session-wise moving window analysis. Windows consisted of 19 trials.—The three experimental sessions are separated by different background colors.
(TIF)

S1 File Behavioral and physiological data archive. This RAR format file includes all analyzed behavioral and physiological data in ASCII format. It also contains the file *Data-Documentation-001.rtf* which explains the layout of the data.
(RAR)
