# Supplementary figures and images for: Improving Accuracy and Temporal Resolution of Learning Curve Estimation for within- and across-Session Analysis
Source: PLoS One. 2016 Jun 15;11(6):e0157355. doi: 10.1371/journal.pone.0157355 (PMC4909298; doi:10.1371/journal.pone.0157355)

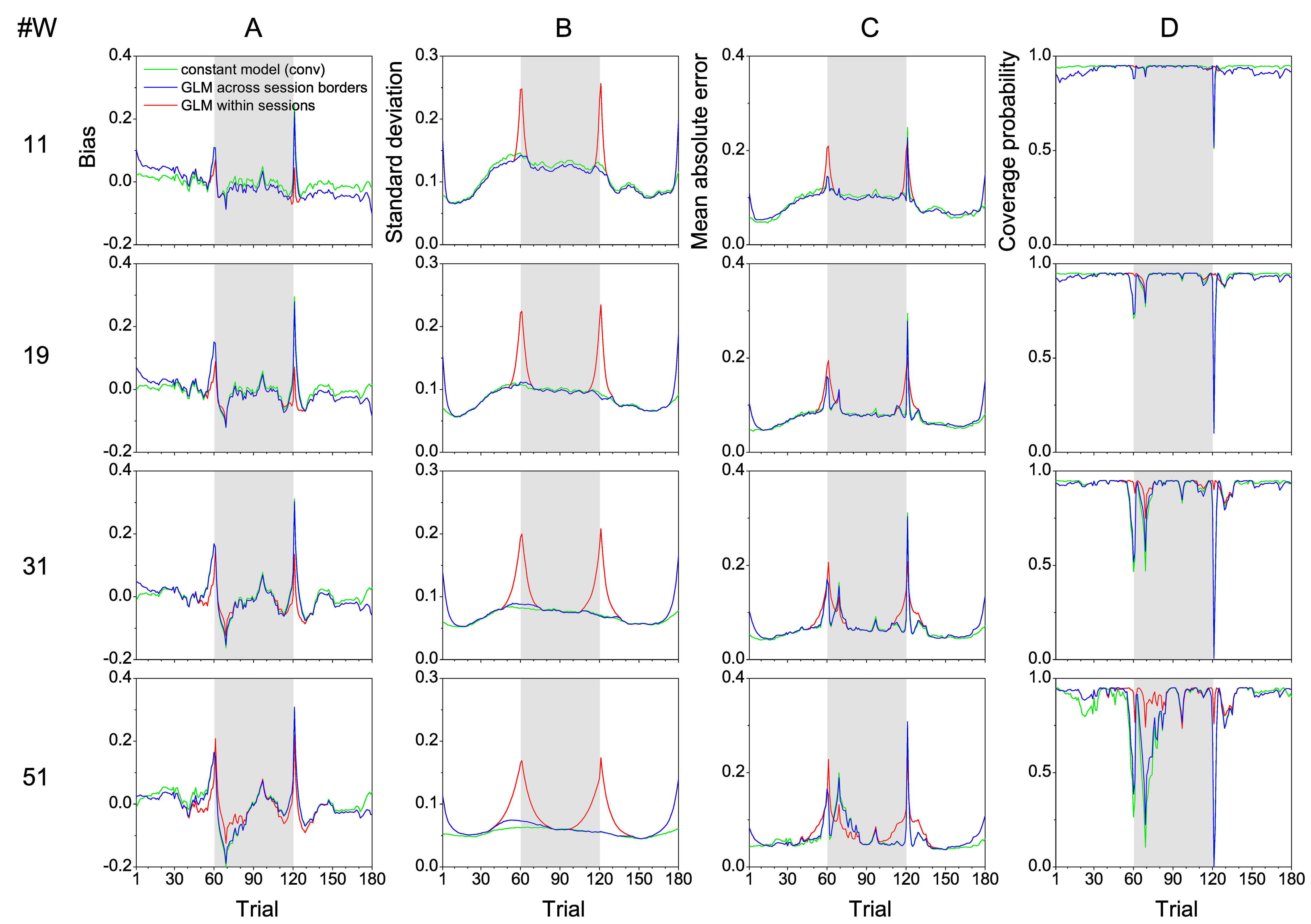

Supplement: S1 Fig — Simulations were carried out as in Fig 4. (A) Bias, (B) standard deviation (SD), (C) mean absolute error (MAE), and (D) coverage probabilities of confidence intervals of the estimated learning curves for four different window sizes (11, 19, 31, and 51). Green and blue curves represent moving window analyses across session borders employing a constant model (conv) and a GLM, respectively. Red curves show results of a GLM with session-wise moving window analysis where session breaks have been taken into accout. Similar systematic errors (bias and low coverage probabilities) are found for the two moving window analyses not accounting for session breaks.—The three experimental sessions are separated by different background colors. (TIF) [file pone.0157355.s001.tif]

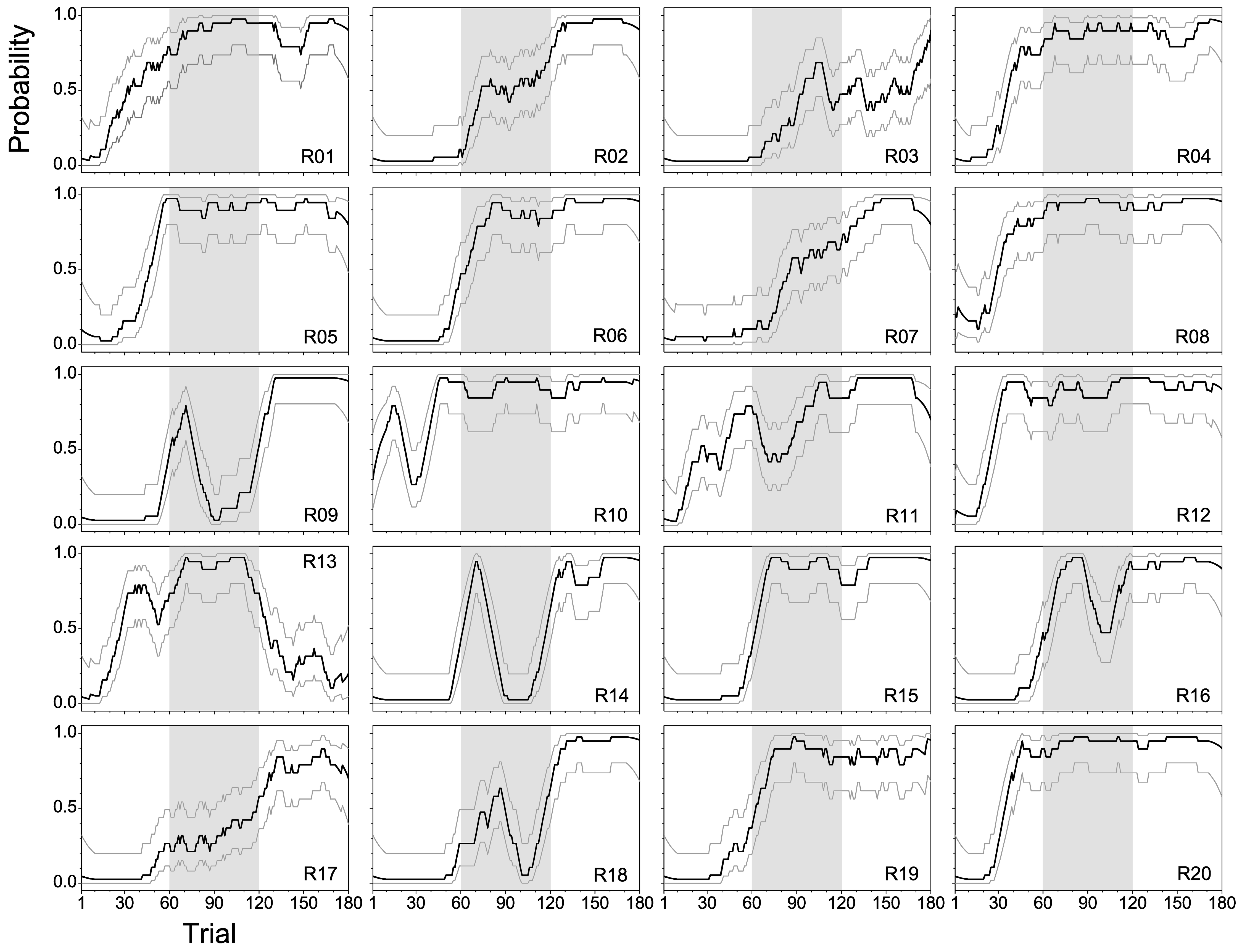

Supplement: S2 Fig — Learning curves (black lines) for the 20 individual rodents (R01 to R20) with 95%-confidence intervals (thin grey lines) derived from a constant model (conv) with a window of 19 trials moving across trials and session borders.—The three experimental sessions are separated by different background colors. (TIF) [file pone.0157355.s002.tif]

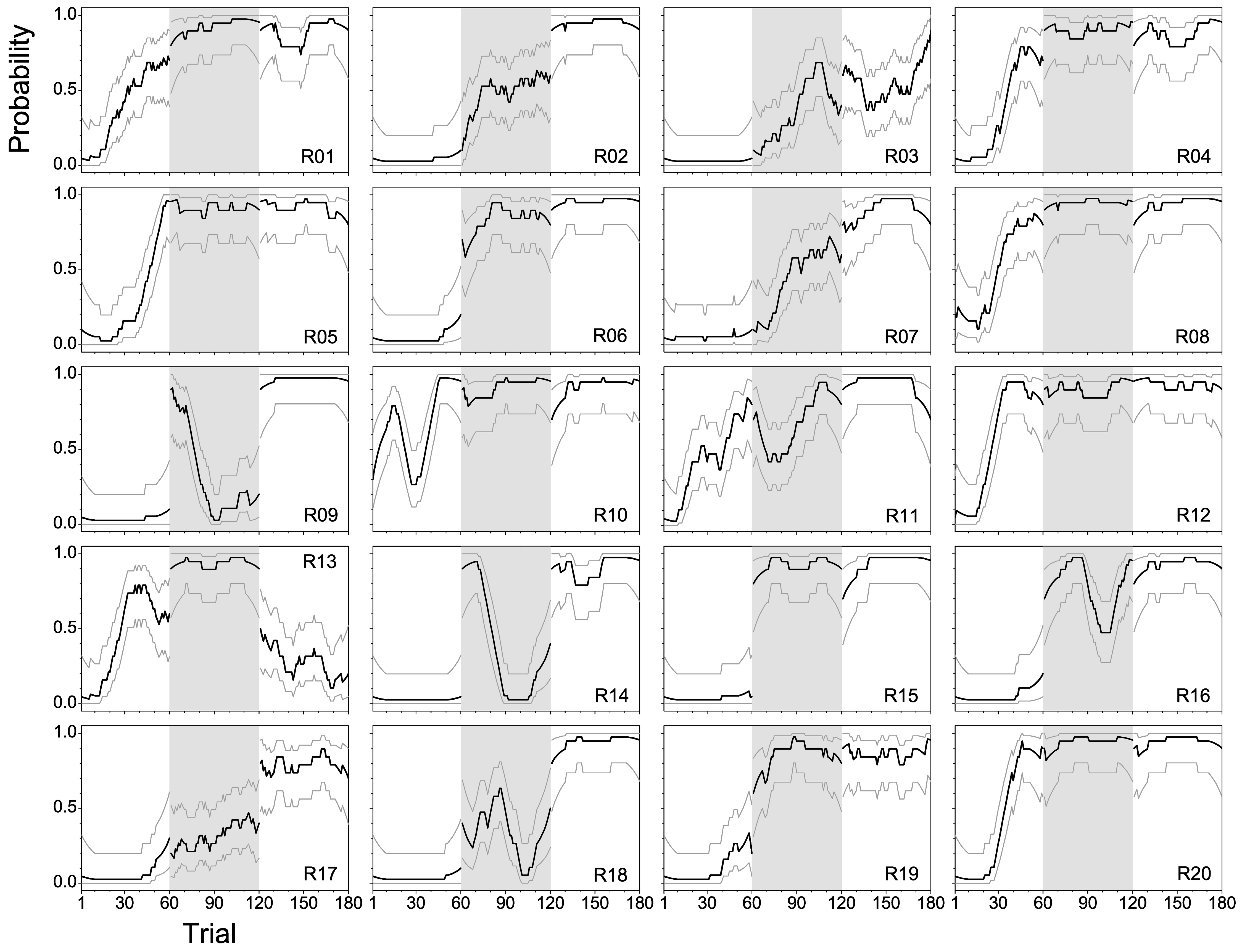

Supplement: S3 Fig — Learning curves (black lines) for the 20 individual rodents (R01 to R20) with 95%-confidence intervals (thin grey lines) derived from a constant model employed in a session-wise moving window analysis. Windows consisted of 19 trials.—The three experimental sessions are separated by different background colors. (TIF) [file pone.0157355.s003.tif]
